# Supplementary material for: Splice-Junction-Based Mapping of Alternative Isoforms in the Human Proteome
Source: Cell Rep. Author manuscript; Available in PMC 2020 Jan 15. (PMC6961840; doi:10.1016/j.celrep.2019.11.026)
Supplement: 2 [file NIHMS1546469-supplement-2.zip › DF1/PXD010154/Ovary/Ovary_9_RPS15_GPRPAAGHVLAEVEQK.pdf]

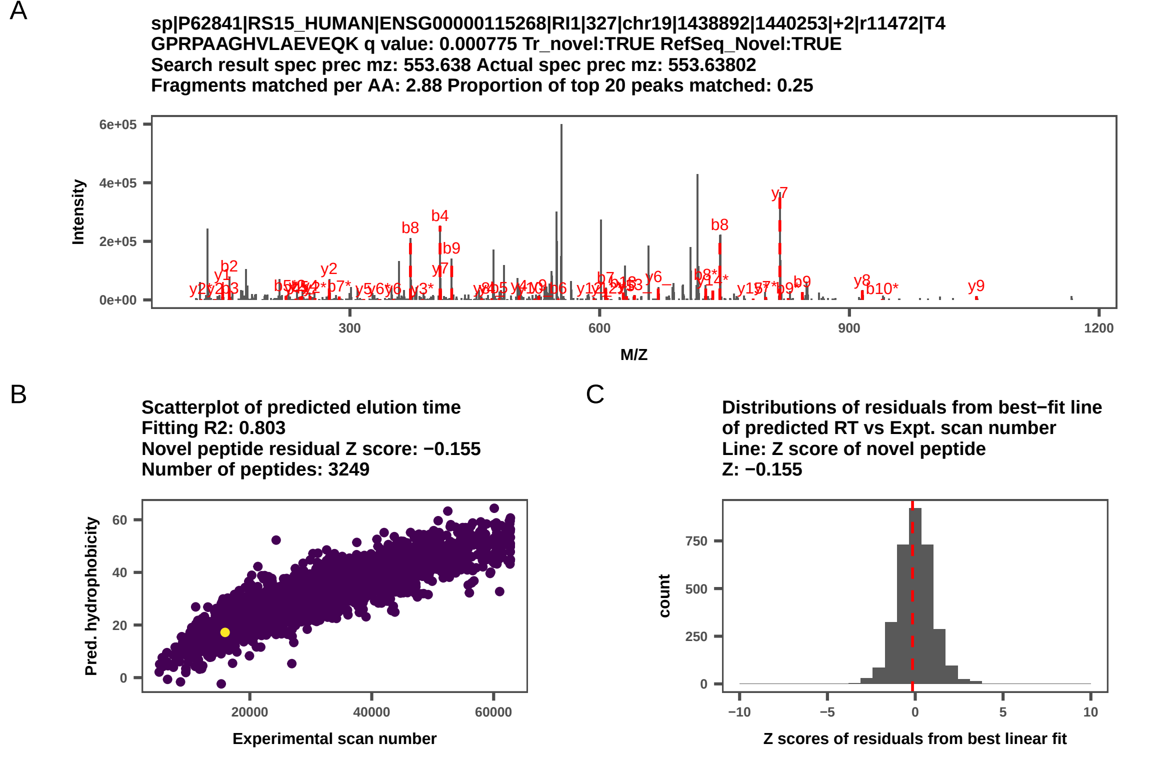
A
sp|P62841|RS15_HUMAN|ENSG00000115268|RI1|327|chr19|1438892|1440253|+2|r11472|T4
GPRPAAGHVLAEVEQK q value: 0.000775 Tr_novel:TRUE RefSeq_Novel:TRUE
Search result spec prec mz: 553.638 Actual spec prec mz: 553.63802
Fragments matched per AA: 2.88 Proportion of top 20 peaks matched: 0.25
Intensity
6e+05
4e+05
2e+05
0e+00
300
600
900
1200
M/Z
y7
b4
b8
b8
b9
y7
b2
y1
y2
y6
b9
y8
b10*
y9
B
Scatterplot of predicted elution time
Fitting R2: 0.803
Novel peptide residual Z score: −0.155
Number of peptides: 3249
Pred. hydrophobicity
60
40
20
0
20000
40000
60000
Experimental scan number
C
Distributions of residuals from best−fit line
of predicted RT vs Expt. scan number
Line: Z score of novel peptide
Z: −0.155
count
750
500
250
0
−10
−5
0
5
10
Z scores of residuals from best linear fit
